# CSNK2B contributes to colorectal cancer cell proliferation by activating the mTOR signaling

**DOI:** 10.1007/s12079-021-00619-1

**Published:** 2021-04-29

**Authors:** Shijun Yu, Qingqing Hu, Kailing Fan, Chen Yang, Yong Gao

**Affiliations:** 1 Department of Oncology, Shanghai East Hospital, Tongji University School of Medicine, 150 Ji-Mo Road, Shanghai, 200120 People’s Republic of China; 2 Center for Disease Control and Prevention, Pudong New Area, Shanghai, 200136 People’s Republic of China; 3 Fudan University Pudong Institute of Preventive Medicine, Pudong New Area, Shanghai, 200136 People’s Republic of China

**Keywords:** *CSNK2B*, *mTOR*, Cell proliferation, Tumorigenesis, Colorectal cancer

## Abstract

The function of Casein kinase 2 beta (*CSNK2B*) in human malignancies has drawn increasing attention in recent years. However, its role in colorectal cancer (CRC) remains unclear. In the present study, we aimed to explore the expression and biological functions of *CSNK2B* in CRC. Public gene expression microarray data from online database and immunohistochemistry analysis demonstrated that *CSNK2B* was highly expressed in CRC tissues than in normal tissues. In vitro and in vivo cellular functional experiments showed that increased *CSNK2B* expression promoted CRC cell viability and tumorigenesis of CRC. Further western blots and rescue experiments confirmed that *CSNK2B* promoted CRC cell proliferation mainly by activating the *mTOR* signaling pathway. These findings identified *CSNK2B* as a novel oncogene contributing to the development of CRC.

**Supplementary Information:**

The online version contains supplementary material available at 10.1007/s12079-021-00619-1.

## Introduction

Colorectal cancer (CRC) is the third most common malignancy worldwide with increasing incidence annually (Bray et al. 2018; Siegel et al. 2020). Although combined therapies including surgical resection, chemotherapy, targeted therapy and radiotherapy have effectively prolonged the overall survival (OS) of patients with early CRC, the long-term prognosis of patients with advanced CRC remains poor due to postoperative recurrence and high metastatic potential of this disease (Aparicio et al. 2020; Shuford et al. 2020). Therefore, a better understanding of the mechanisms underlying CRC tumorigenesis and progression is critical for developing effective therapeutic strategies.

Casein kinase 2 beta (*CSNK2B*) is a gene encoding the beta subunit of casein kinase II (*CK2*) (Heller-Harrison et al. 1989), a ubiquitous protein kinase which regulates a plethora of biological functions including cell proliferation, differentiation and survival by phosphorylating serine and threonine residues of its target proteins (Litchfield 2003; Silva-Pavez and Tapia 2020). *CK2* is composed of 2 alpha subunits and a dimer of beta subunits. While the catalytic subunit of *CK2* controls the kinase activity of *CK2*, *CSNK2B*, is thought to regulate the substrate-specific targeting of catalytic subunits (Guerra and Issinger 1999).

In the area of cancer research, emerging evidence has gradually suggested a critical role of *CSNK2B* in various malignancies (Filhol et al. 2015; Pallares et al. 2009). For example, overexpression of *CSNK2B* has been found to activate the *NF-κB* signaling in hepatocellular carcinoma (HCC), thus promoting cell proliferation and inhibiting cell apoptosis of HCC (Xiao et al. 2020). In another study of breast cancer (BC), higher *CSNK2B* expression was significantly correlated with worse prognosis of patients suffering from BC (Wang et al. 2019). However, the role of *CSNK2B*, and its mechanism of functions in CRC are still obscure.

In the present study, we resorted to explore the expression patterns and functions of *CSNK2B* in CRC. Our results indicated that *CSNK2B* expression is frequently over-expressed in CRC tissues than in normal tissues, and *CSNK2B* promotes CRC cell proliferation in vitro and in vivo via activating the *mTOR* signaling pathway.

## Materials and methods

### Cell culture and reagents

Human CRC cell lines HT29, LoVo, DLD1, SW620, SW480, Caco-2, HCT116 and RKO were purchased from Shanghai Cell Bank of Chinese Academy of Science (Shanghai, China). Cell line authenticity and mycoplasma contamination were routinely checked by PCR-based assays and STR genotyping, respectively, in 6–10-month intervals. Cells were cultured with Dulbecco's modified Eagle medium (DMEM; Gibco, USA) supplemented with 10% fetal bovine serum (FBS; Gibco, USA) and 1% penicillin/streptomycin (#15140122, Thermo Fisher Scientific, USA) in a humidified incubator with 5% CO_2_ at 37 °C. All cell line experiments were conducted within 10 passages after thawing. Rapamycin was supplied by Selleck Chemicals (#S1039, Shanghai, China), and the working concentration was 20 nM.

### Immunohistochemical analysis

To evaluate *CSNK2B* protein expression in CRC, a tissue microarray (TMA) containing 50 paired CRC tumor tissues and adjacent normal tissues was obtained from Alenabio Biotech Company (#DC-Col02006, Xian, China), and immunohistochemical (IHC) analysis was performed following standard staining procedures with a specific antibody against *CSNK2B* (1:200, #20234-1-AP, ProteinTech, Wuhan, China). Briefly, the paraffin-embedded sections were deparaffinized by xylene, followed by re-hydration using graded ethanol and then rinsed with deionized water. 3% hydrogen peroxide was used to block endogenous peroxidase activity. For antigen retrieval, the sections were high-pressure-treated and boiled in a 10 mM citrate buffer (pH 6.0) for 4 min. Nonspecific binding was blocked with 5% normal goat serum for 30 min at room temperature. After which, the sections were incubated with primary antibodies overnight at 4℃ and subjected to incubation with horseradish peroxidase-conjugated secondary antibody. Finally, the sections were stained with the DAB substrate at room temperature. The final IHC scores were analyzed by two pathologists blinded to the clinical information independently based on the percentage of stained cells and staining intensity. Briefly, the percentage of CSNK2B-positive cells was classified into 5 groups: < 10% (0), 10–25% (1), 25–50% (2), 50–75 (3), and > 75% (4). The staining intensity was divided into 4 groups: no staining (0), light brown (1), brown (2), and dark brown (3). The overall IHC scores of CSNK2B were calculated using the following formula: overall score = percentage score × intensity score. The samples with an overall score ≤ 6 were defined as weak staining, and > 6 were defined as strong staining. Ethical approval was granted by the ethics committee of Shanghai East Hospital, Tongji University School of Medicine, China.

### Online gene expression analysis of gene expression omnibus datasets

To verify the expression of *CSNK2B* in CRC tissues, previously published microarray data from Gene Expression Omnibus (GEO) datasets (GEO accession number: GSE6988, GSE8671 GSE20842 and GSE20916) were reanalyzed using GEO2R, a R-based publicly online tool for analyzing GEO-deposited gene expression data (http://www.ncbi.nlm.nih.gov/geo/geo2r).

### Plasmid and small-inference RNA transfection

*CSNK2B*-expressing plasmid containing a Flag tag (p*CSNK2B*, GeneBank Accession Number: NM_001320) was purchased from Vigene Biosciences, Shandong, China (#CH817776), and small inference RNAs (siRNAs) specifically against *CSNK2B* (si*CSNK2B*-1 and si*CSNK2B*-2) were designed and synthesized by GenePharma, an empty plasmid pcDNA3.1 (pVEC) and negative control siRNA (siNC) served as their control groups, respectively. Cell transfection was performed using Lipofectamine 3000 Transfection Reagent (Invitrogen, CA, USA) in accordance with the instructions. To generate stable *CSNK2B*-overexpression or knockdown cell lines, human embryonic kidney (HEK)-293T cells were used to package the recombinant lentivirus containing *CSNK2B* gene and shRNAs against *CSNK2B* (sh*CSNK2B*). The CRC cell lines were infected with lentivirus particles in the presence of Polybrene (4 μg/ml). After 48 h, puromycin (2.5 μg/ml, Sigma, MO, USA) was added to the medium to select stably infected cells. The sequences of siRNAs are as follows (5′–3′): si*CSNK2B*-1: CUCCGUGGCAAUGAAUUCUdTdT, si*CSNK2B*-2: GUCAAGACGAUUCGCUGAUdTdT, siNC: UUCUCCGAACGUGUCACGUdTdT, among which si*CSNK2B*-2 was chosen to construct sh*CSNK2B*.

### Western blot analysis

Total cellular proteins were extracted using RIPA lysis buffer (#20-188, Millipore, USA) containing protease inhibitor cocktail (#P8340, Signa-Akdrich, USA) and phosphatase inhibitor cockatil (#P2850, Signa-Akdrich, USA), protein concentration was determined with BCA Protein Assay Kit (#23225, Thermo Fisher Scientific, USA) according to the manufacturer’s protocol. Protein samples were boiled in 1× sodium dodecyl sulfate polyacrylamide gel electrophoresis (SDS-PAGE) loading buffer (#KGP101, KeyGENBio, Nanjing, China) and equal amounts of them were subjected to 10% SDS-PAGE (30 μg of protein were loaded per lane, 5% laminating gel, 10% separation gel, laminated glue voltage 80 V, separation gel voltage 150 V, electrophoresis time 60 min) followed by transfer onto a nitrocellulose membrane (1 h transfer at constant current of 250 mA). The membrane was then soaked in 5% non-fat milk for 1 h to block unspecific binding and subjected to incubation with diluted primary antibodies overnight at 4 °C, after which the membrane was incubated with secondary antibodies for 1 h at room temperature. The bands were visualized using a LI-COR Odyssey imaging system (LI-COR Biosciences, USA). Specific antibodies used in this study are as follows: anti-*CSNK2B* (1:2000, #20234-1-AP, ProteinTech, Wuhan, China), anti-Flag tag (1:1000, #abs137958 Absin Bioscience, Shanghai, China), anti-*β-actin* (1:500, #sc-47778, Santa Cruz Biotechnology, CA, USA), anti-*mTOR* (1:1000, #2983S, Cell Signaling Technology, MA, USA), anti-P-*mTOR* (1:1000, #5536S, Cell Signaling Technology, MA, USA), anti-*STAT3* (1:1000, #9139S, Cell Signaling Technology, MA, USA), anti-P-*STAT3* (1:1000, #9145S, Cell Signaling Technology, MA, USA), anti-*FAK* (1:500, # 12636-1-AP, ProteinTech, Wuhan, China), anti-p53 (1:200, #sc-126, Santa Cruz Biotechnology, CA, USA), anti-*cyclin D1* (1:800, #55506S, Cell Signaling Technology, MA, USA), anti-*CDK4* (1:500, #11026-1-AP, ProteinTech, Wuhan, China), anti-*NRF2* (1:500, # 16396-1-AP, ProteinTech, Wuhan, China), anti-*p70S6K* (1:1000, #2708S, Cell Signaling Technology, MA, USA), anti-P-*p70S6K* (1:500, #9234, Cell Signaling Technology, MA, USA), anti-*4E-BP1* (1:800, #9644, Cell Signaling Technology, MA, USA) and anti-P-*4EBP1* (1:500, #2855, Cell Signaling Technology, MA, USA). Immunoblots shown are representative of at least three independent biological replicates.

### Cell proliferation assay

CRC cell proliferation abilities were assessed using Cell Counting Kit-8 (CCK-8) (#CK04, Dojindo Laboratories, Kumamoto, Japan). Briefly, the indicated CRC cell lines were seeded into a 96-well plate with a density of 3 × 10^3^ cells/well. After adherence, 10 μl of CCK-8 reagent was added into each well at the corresponding time points (0, 1, 2, 3, 4 and 5 days) and incubated for 1 h. Values were acquired as absorbance at 450 nm on a SpectraMax M5 microplate reader (Molecular Device, CA, USA). Each experiment was done in triplicate and repeated at least 3 times independently.

### Colony formation assay

Colony-forming capacities were evaluated by colony formation assays. Briefly, the indicated CRC cell lines were seeded into a 6-well plate with a density of 1 × 10^3^ cells/well and cultured for 2 weeks under normal conditions. Subsequently, formed colonies were fixed using 4% paraformaldehyde for 15 min and stained using 0.5% crystal violet for 10 min at room temperature. The stained colonies were then photographed and counted using Image J software.

### EdU incorporation assay

EdU incorporation assay was conducted using an EdU labeling kit (#C10310-1, RiboBio, Guangzhou, China) according to the manufacturer’s instructions. In brief, cells were seeded into a 24-well plate with cell density at 60% and incubated for 24 h. Next, 50 μM of EdU was added into each well and incubated for 2 h. After fixation with 4% paraformaldehyde, incubation with glycine (2 mg/ml) and subsequent incubation with 1× Apollo reaction cocktail, the plate was protected from light and stained with Hoechst 33342 (5 μg/ml) for 30 min at room temperature. Finally, the stained cells were visualized under a fluorescence microscope (Leica Microsystems, Wetzlar, Germany). 5 fields were randomly selected and the EdU incorporation rates were quantified as the ratio of EdU-positive cells (red) to Hoechst 33342-positive cells (blue).

### Animal experiments

4–6 weeks old BALB/c nude mice were purchased from SLAC Laboratory Animal Co., Ltd., Shanghai, China. A subcutaneous xenograft tumor model of nude mice was established for in vivo tumor growth assays. 2 × 10^6^ HCT116 cells stably overexpressing *CSNK2B* and SW620 cells with stable knockdown of *CSNK2B* were inoculated subcutaneously into left or right flank of nude mice (n = 6), respectively. After 3 (HCT116) or 5 (SW620) weeks, the nude mice were euthanized, tumors were removed, photographed and weighted. The nude mice were housed in the SPF animal laboratory at Tongji University, which provided a 12-h light/dark cycle and nude mice were allowed ad libitum access to food and water. All animal handling and experimental procedures were abided by the Ethics Committee of Shanghai East Hospital, Tongji University School of Medicine (Authorization number: 2019tjdx62) and in accordance with the ARRIVE guidelines (https://www.nc3rs.org.uk/arrive-guidelines).

### Statistical analysis

Data were presented as mean ± standard deviation (SD) for three biological replicates for all experiments, and statistical analyses were performed using GraphPad Prism software v7.0. Gaussian distribution data were analyzed by two-tailed Student’s t test, while non-Gaussian distribution data by Mann–Whitney of nonparametric test, and one-way ANOVA with a post-hoc Turkey test was used for multiple comparisons, and *P* < 0.05 was considered to indicate a statistically significant difference.

## Results

### *CSNK2B* expression levels were frequently increased in CRC tissues

**Fig. 1:**
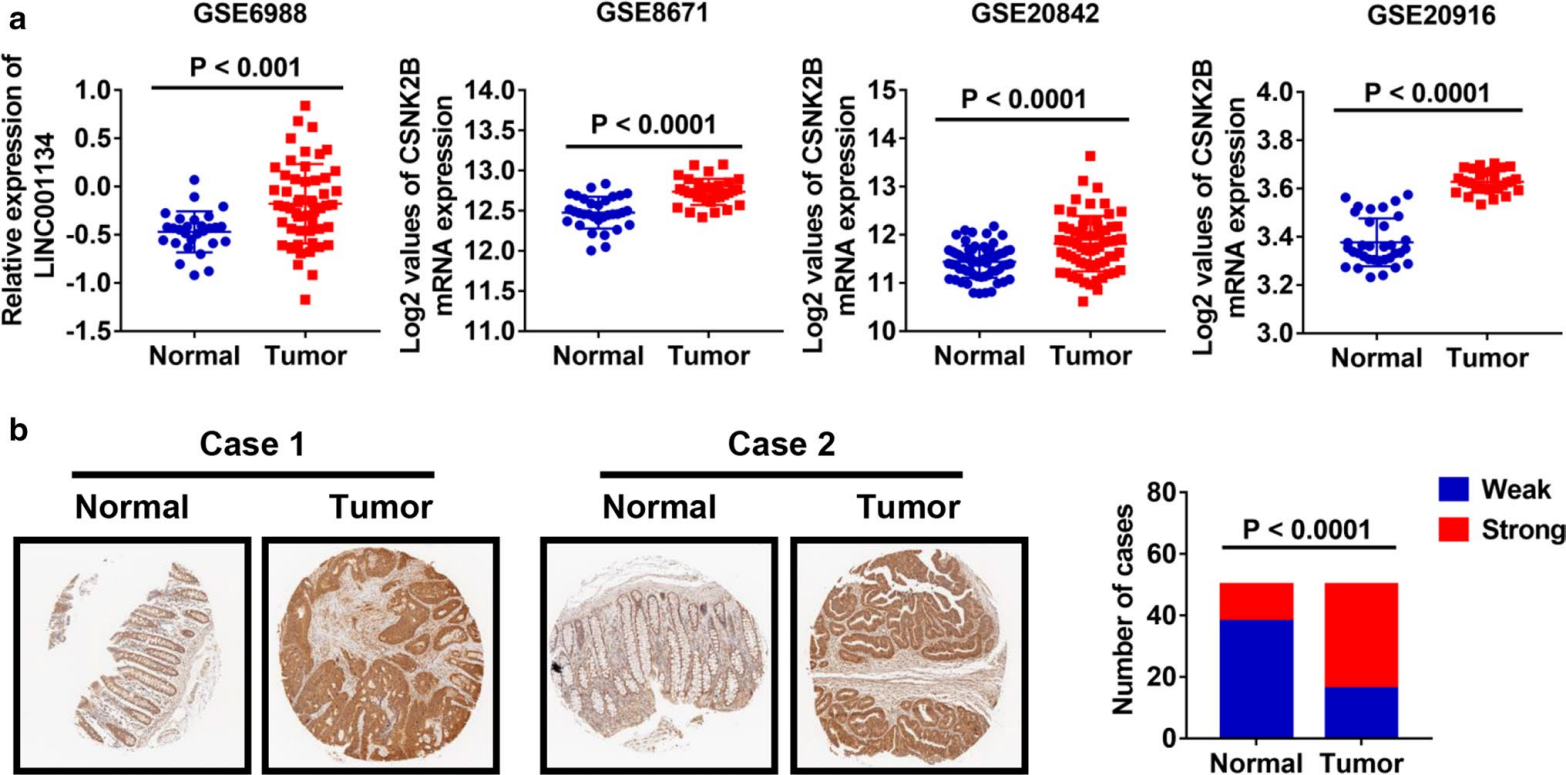
*CSNK2B* expression was significantly up-regulated in CRC tissues. **a** Human CRC gene expression data from public GEO database (GSE6988, GSE8671 GSE20842 and GSE20916) were analyzed to compare *CSNK2B* mRNA level between tumor and normal tissues. **b** *CSNK2B* protein level in CRC was detected by IHC analysis on a tissue microarray containing 50 CRC tissues and paired adjacent normal tissues. Representative images (left panel, magnification: ×40) and column diagram (right panel) were shown

To explore the role of *CSNK2B* in CRC, we firstly analyzed *CSNK2B* mRNA expression utilizing 4 previously published gene expression profiles from GEO database (GSE6988, GSE8671 GSE20842 and GSE20916). As shown in Fig. 1a, increased *CSNK2B* mRNA level was observed in all CRC tissues compared with normal tissues. Furthermore, *CSNK2B* protein expression was evaluated by IHC method on a CRC tissue microarray containing 50 CRC tissues and paired adjacent normal tissues. Consistently, *CSNK2B* protein level in CRC tissues was also significantly higher than in normal tissues (Fig. 1b, *P* < 0.0001). Therefore, these data suggested an overall up-regulation of *CSNK2B* mRNA and protein levels in CRC tissues, hinting us that *CSNK2B* is possibly involved in the development of CRC.

### Overexpression of *CSNK2B* accelerated CRC cell proliferation in vitro

**Fig. 2:**
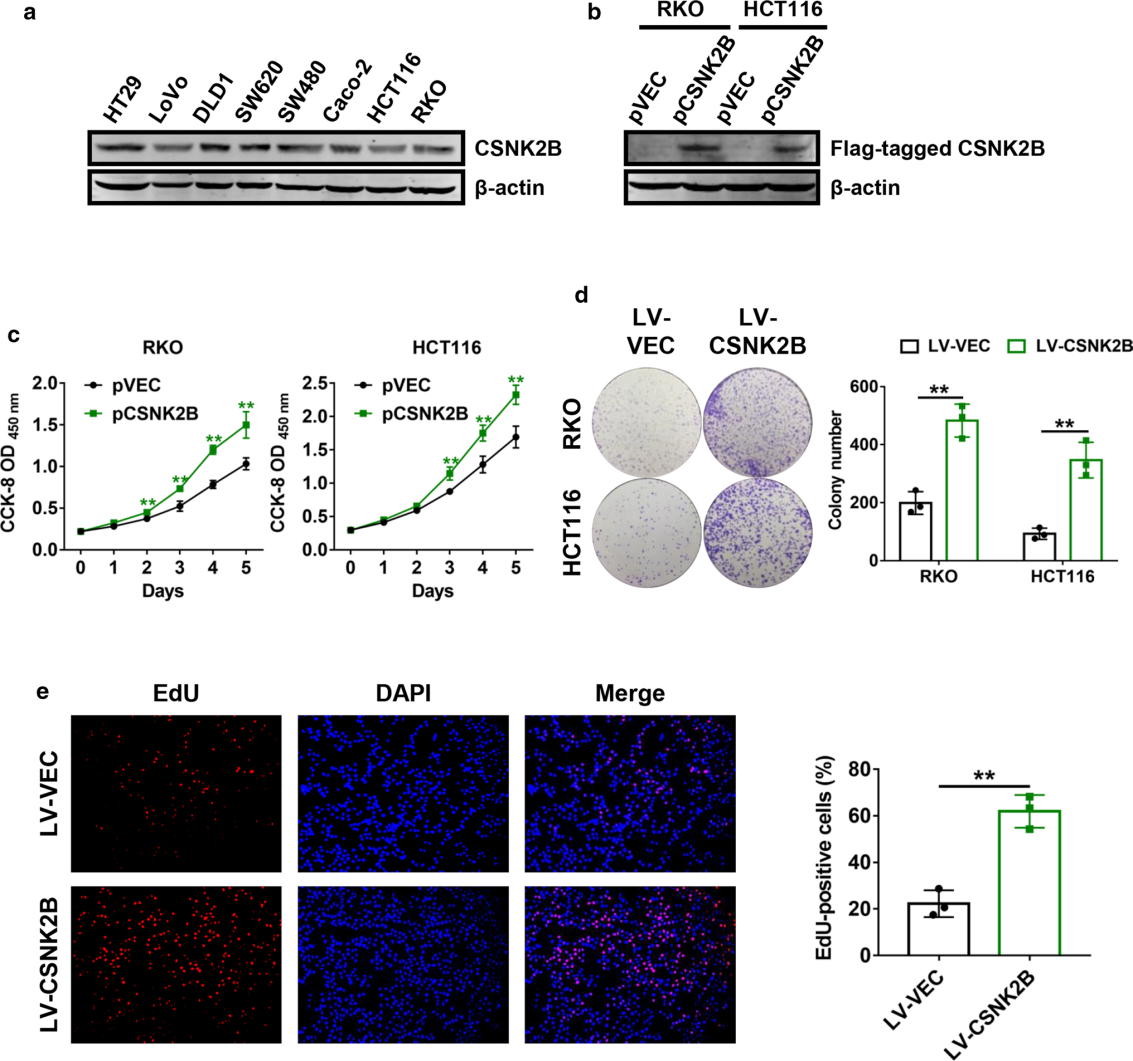
Overexpression of *CSNK2B* promoted CRC cell proliferation in vitro. **a** *CSNK2B* protein expression in different CRC cell lines was evaluated by western blot analysis. **b** RKO and HCT116 cells were transfected with Flag-tagged *CSNK2B*-expressing plasmids, total proteins were extracted after 24 h and western blot analysis was performed. **c** CCK-8 cell proliferation assays were employed at 24 h post-transfection of *CSNK2B*-expressing plasmids into RKO and HCT116 cells. **d** RKO and HCT116 cells with stable *CSNK2B* overexpression were used to perform colony formation assays. Representative images (left panel) and quantification (right panel) of colonies were shown. **e** Representative images (left panel) and quantification (right panel) of EdU-positive RKO cells were shown. *P < 0.05, **P < 0.01

We next investigated whether *CSNK2B* plays a role in CRC progression. Endogenous *CSNK2B* expression in different CRC cell lines was analyzed using western blot analysis. As shown in Fig. 2a, *CSNK2B* was present in all of the analyzed cell lines. To assess the function of *CSNK2B* in CRC cell proliferation, RKO and HCT116 with a relatively low level of *CSNK2B* were transiently transfected with *CSNK2B*-expressing plasmids (Fig. 2b), and CCK-8 cell proliferation assays were performed. The growth curves suggested that ectopic expression of *CSNK2B* significantly facilitated CRC cell growth rates (Fig. 2c). Besides, we generated stable *CSNK2B*-overrexpressing RKO and HCT116 cells with lentivirus-mediated transduction and carried out colony formation assays. As expected, the number of colonies formed from cells with overexpression of *CSNK2B* was remarkably higher than those from the control cells (Fig. 2d). In addition, EdU incorporation assay was performed to verify the effects of *CSNK2B* on CRC cell proliferation. Similarly, *CSNK2B*-overexpressing RKO and HCT116 cells exhibited a higher percentage of EdU-positvie cells, which further supported the above observations (Fig. 2e). Hence, these findings demonstrated that increased *CSNK2B* expression strongly enhanced CRC cell proliferation abilities.

### Negative regulation of cell growth by *CSNK2B* knockdown in vitro

**Fig. 3:**
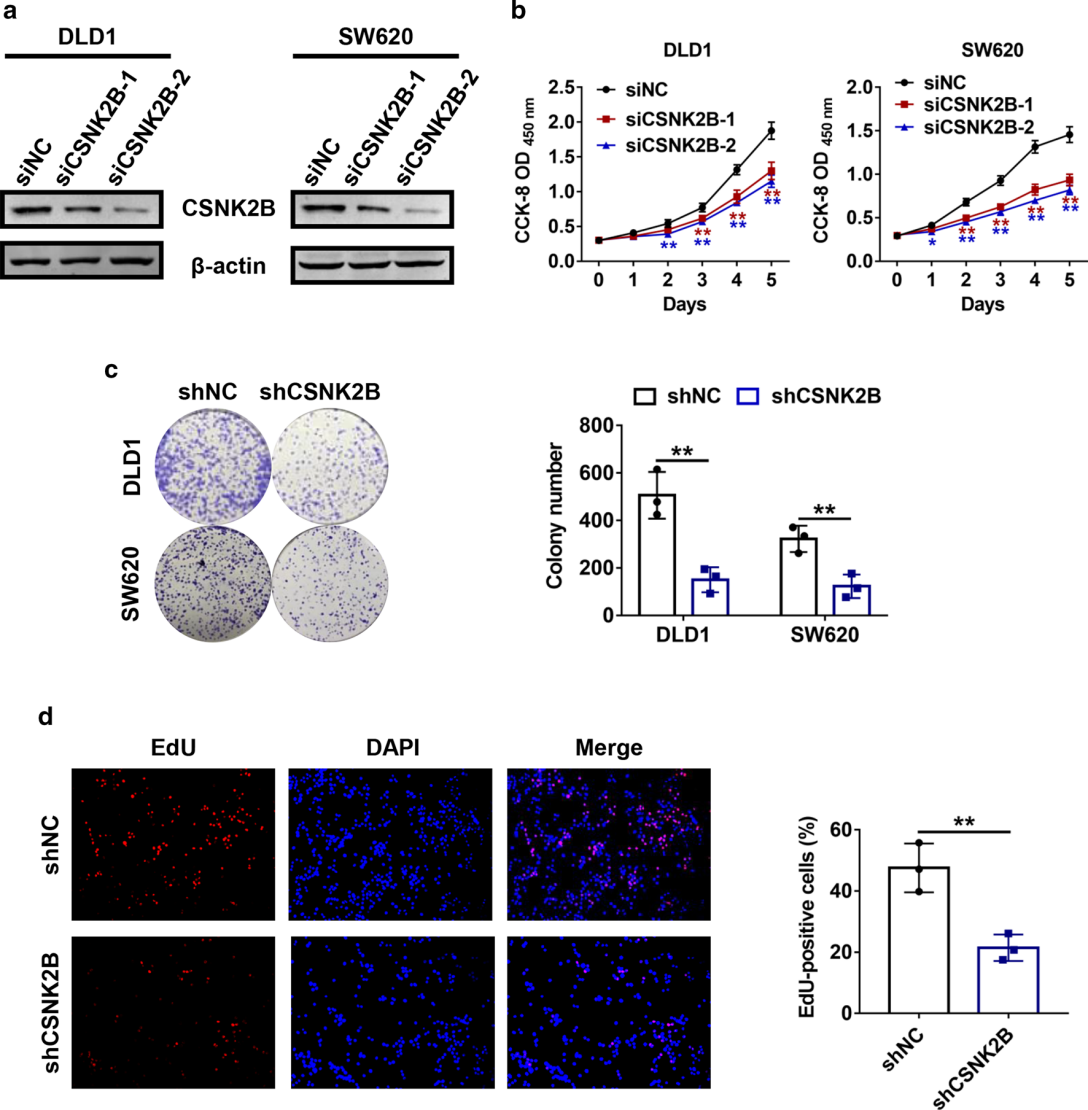
Knockdown of *CSNK2B* retarded CRC cell viability in vitro. **a** DLD1 and SW620 cells were transfected with siRNAs against *CSNK2B*, respectively. 48 h after transfection, cells were lysed and immunoblots were performed. **b** Cell growth curves were measured using CCK-8 assays after 24 h post-transfection of the indicated siRNAs. **c** Representative images (left panel) and quantification (right panel) of colonies formed from DLD1 and SW620 cells with stable knockdown of *CSNK2B* were exhibited. **d** Relative proliferation rates of the indicated cells were assessed by EdU staining, and representative images (left panel) and counting analysis (right panel) were shown. *P < 0.05, **P < 0.01

Subsequently, whether down-regulated *CSNK2B* expression could affect proliferation capacities of the CRC cells were explored. DLD1 and SW620 cells with a relatively higher *CSNK2B* expression level were transfected with siRNAs against *CSNK2B* (si*CSNK2B*-1 and si*CSNK2B*-2), and immunoblots confirmed the knockdown efficiencies (Fig. 3a). In contrast to overexpression, blockade of *CSNK2B* markedly retarded cell growth rates comparing with the corresponding control groups (Fig. 3b). Moreover, DLD-1 and SW620 with stable knockdown of *CSNK2B* using lentiviral infection of shRNA were established, and concordant results were observed in colony formation assays (Fig. 3c). Moreover, *CSNK2B*-knockdown cells exhibited a significantly lower percentage of EdU-positive cells in EdU incorporation assays (Fig. 3d). Taken together, these in vitro observations identified that *CSNK2B* plays an oncogenic role in CRC cell proliteration in vitro.

### *CSNK2B* promoted CRC tumor growth in vivo

**Fig. 4:**
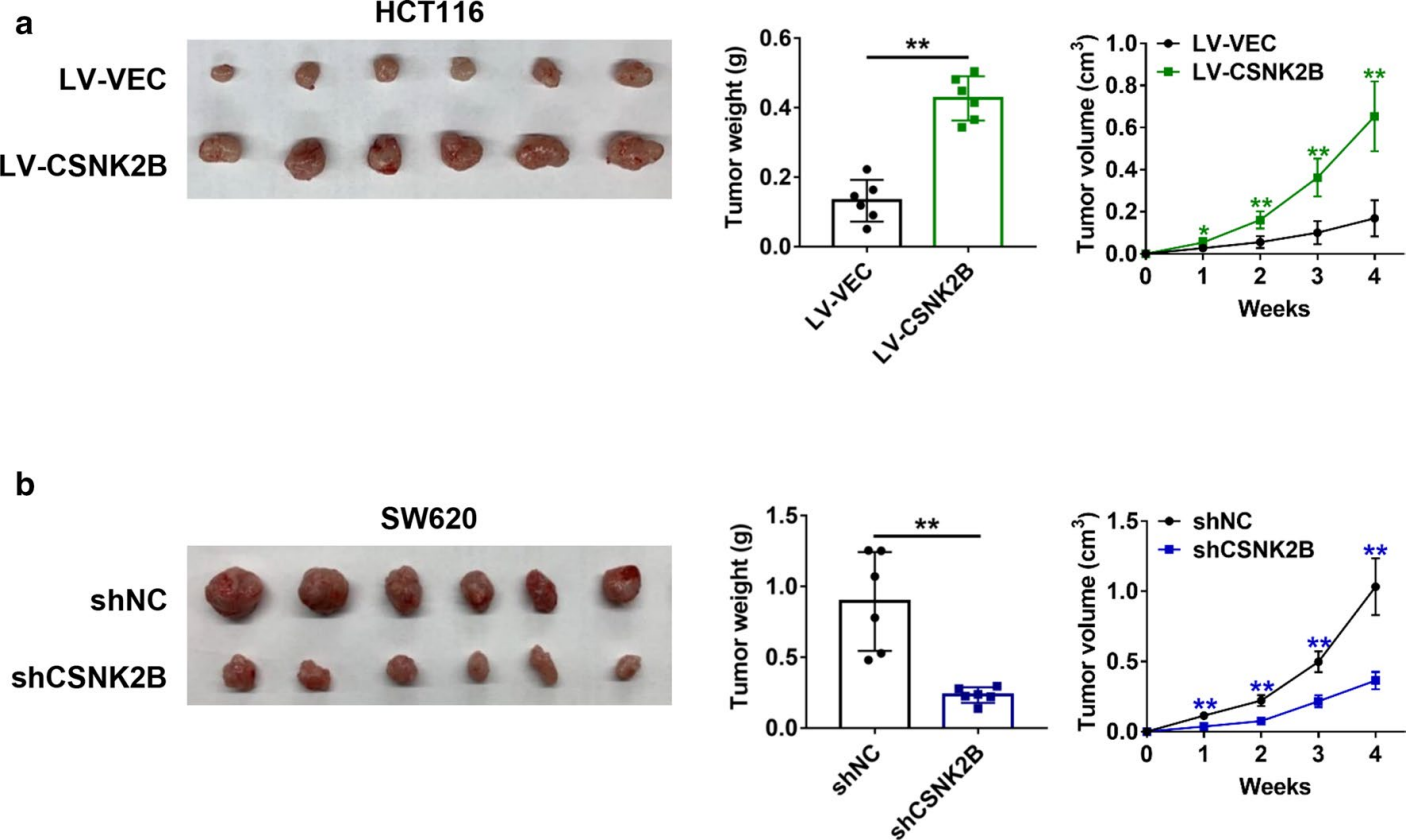
CRC tumor growth was positively regulated by *CSNK2B*. **a**–**b** A subcutaneous xenograft model of nude mice was established using HCT116 cells with stable *CSNK2B* overexpression (**a**) or SW620 cells with stable *CSNK2B* knockdown (**b**) (n = 6). Xenograft tumor images (left panel), tumor volume curves (middle panel) and tumor weight quantification (right panel) were shown. **P < 0.01

With the aim of verifying the role of *CSNK2B* in CRC tumorigenesis in vivo, we established a subcutaneous xenograft mice model by inoculation of the CRC cells with stable overexpression or knockdown of *CSNK2B*. As shown in Fig. 4a, increased *CSNK2B* expression significantly accelerated tumor growth of HCT116 cells. In sharp contrast, *CSNK2B*-knockdown tumors exhibited a markedly tumor growth rate than the control cells (Fig. 4b). Thus, these above results demonstrated that *CSNK2B* contributes to CRC tumorigenesis in vivo.

### The *mTOR*/*p70S6K* signaling mediated the regulation of CRC proliferation by *CSNK2B*

**Fig. 5:**
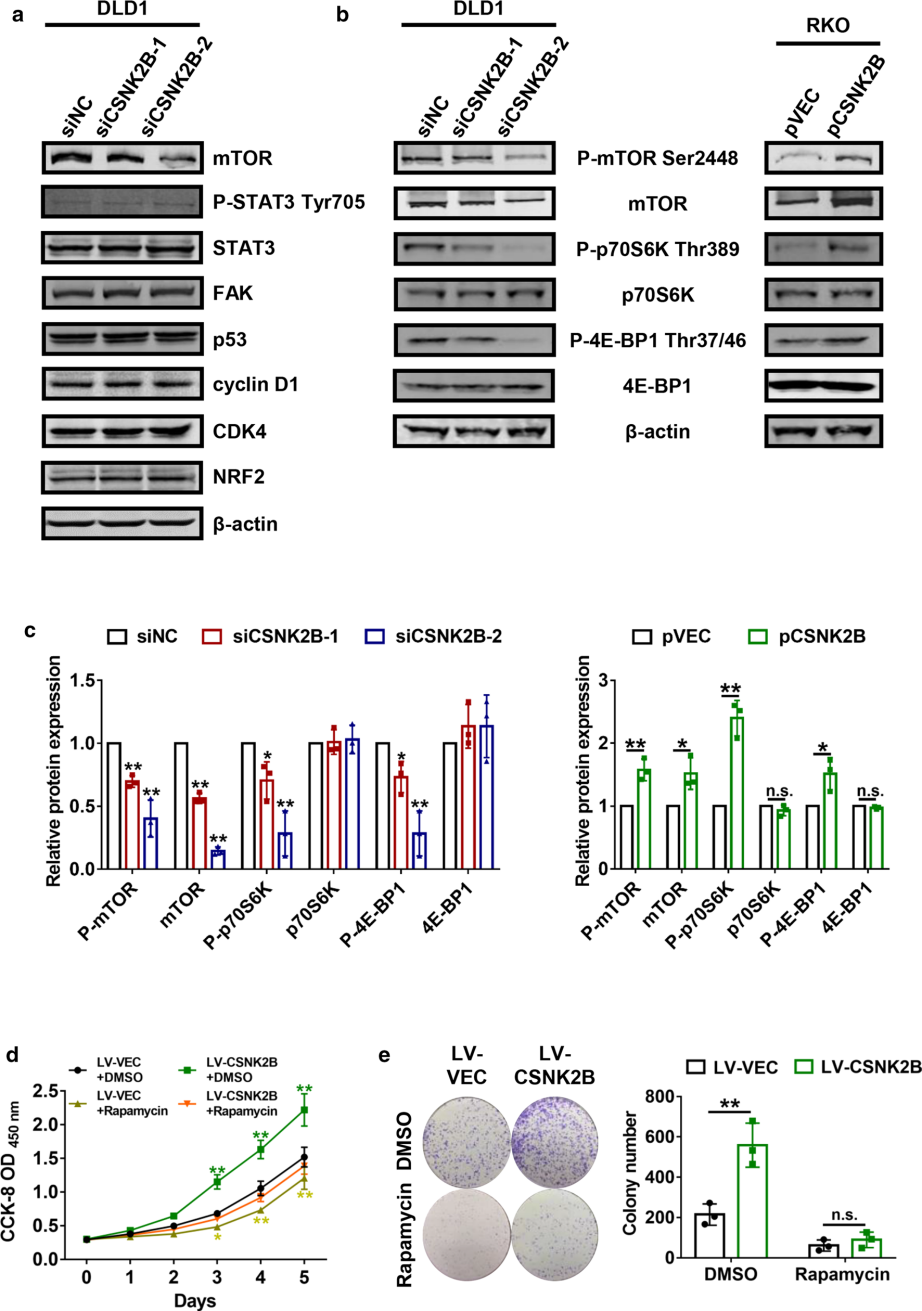
The *mTOR*/*p70S6K* signaling pathway mediated the positive effects of *CSNK2B* on CRC cell proliferation. **a** DLD1 cells were transfected with si*CSNK2B*-1 and si*CSNK2B*-2 for 48 h, then total proteins were extracted and western blot analysis was performed to detect the levels of the indicated genes. **b**–**c** CRC cells transfected with *CSNK2B*-expressing plasmids or siCSNK2B-1/2 were subjected to immunoblots to verify the expression changes of the indicated genes, images (**b**) and densitometric quantification (**c**) were shown. RKO cells with stable *CSNK2B* overexpression were treated with rapamycin (20 nM) or DMSO, CCK-8 assays (**c**) and colony formation assays (**d**) were performed to explore whether *mTOR* inhibition could reverse the effects of *CSNK2B* overexpression on CRC cell viability. *P < 0.05, **P < 0.01

In order to further explore potential mechanisms by which *CSNK2B* affected tumor proliferation, we compared the differential expression of key proteins in crucial tumor-associated pathways including *mTOR* (Murugan 2019), *STAT3* (Garg et al. 2020), *FAK* (Worthmuller and Ruegg 2020), p53 (Huang 2020), *cyclin D1* (Bates and Peters 1995), *CDK4* (Teh and Aplin 2019) and *NRF2* (Liu et al. 2020) by immunoblots after knockdown of *CSNK2B* in DLD1 cells. Interestingly, down-regulation of *CSNK2B* induced a decrease of *mTOR* expression, while the other proteins showed no significant changes (Fig. 5a). It is well established that *mTOR* is a key member of the phosphatidylinositol 3-kinase-associated kinase superfamily and plays an important role in regulating mammalian cell proliferation and survival (Bracho-Valdes et al. 2011; Sun 2020). More importantly, substantial evidence has elucidated that *mTOR* acts as an oncogene in various cancers through activation its downstream molecule such as *p70S6K* and *4E-BP1* (Sridharan and Basu 2020). Thus we subsequently examined the relationship between *CSNK2B* and the activation of the *mTOR* signaling pathway. As expected, decreased phosphorylation levels of *mTOR*, *p70S6K* and *4E-BP1* were observed in DLD-1 cells with knockdown of *CSNK2B*, whereas overexpression of *CSNK2B* led to opposite results (Fig. 5b, c), suggesting that *CSNK2B* can positively regulate the activity of the *mTOR* signaling. Therefore, we hypothesized *CSNK2B* promoted CRC cell proliferation dominantly by activation of the *mTOR* pathway. To verify our hypothesis, CRC cells with stable overexpression of *CSNK2B* were treated with rapamycin, an inhibitor of *mTOR* (Edwards and Wandless 2007). CCK-8 assays showed that the promoting effects of *CSNK2B* on cell proliferation were largely reversed by rapamycin treatment (Fig. 5c), and similar results were observed in colony formation assays (Fig. 5d). Thus, the above findings confirmed that *CSNK2B* promoted CRC cell proliferation via activating the *mTOR* signaling pathway.

## Discussion

Although *CSNK2B* has been implicated in the progression of several human malignancies, its expression patterns and biological function in CRC are extensively unknown. In the current work, we for the first time identified *CSNK2B* as an oncogene in CRC. By analyzing public gene expression data from GEO database, we observed a significant up-regulation of *CSNK2B* mRNA level in CRC tissues comparing with normal tissues, and further IHC analysis showed that *CSNK2B* protein level is up-regulated in CRC. Meanwhile, considering that there is a discrepancy of *CSNK2B* gene expression between different datasets, it is necessary to collect clinical and pathological data to further verify the effectiveness of the above findings and explore whether *CSNK2B* expression is associated with clinicopathological parameters and prognosis of CRC patients in future studies.

Subsequently, we conducted a range of functional experiments to investigate the role of *CSNK2B* in proliferation ability of CRC cells. Both in vitro and in vivo experiments indicated that *CSNK2B* potentiated CRC cell proliferation and tumorigenesis. By mechanistic studies, we found that the effects of *CSNK2B* on CRC cell proliferation were dominantly mediated by abnormal activation of the *mTOR* signaling pathway, which thus contributing to CRC progression.

Currently, *mTOR* is commonly recognized as a key regulator of cell proliferation and survival by stimulating two major downstream targets *p70S6K* and *4E-BP1* (Bracho-Valdes et al. 2011; Sridharan and Basu 2020). Abnormal activation of the *mTOR* signaling pathway is correlated with the progression of different tumor types including CRC (Ghanaatgar-Kasbi et al. 2019; Lu et al. 2020; Murugan 2019). To date, the regulatory relationship between *CSNK2B* and the *mTOR* signaling has not been reported. Herein, we reported that up-regulated *CSNK2B* expression can result in activation of *mTOR* and its downstream effectors *p70S6K* and *4E-BP1*, and inhibition of *mTOR* activity by rapamycin treatment significantly abolished the positive effects of *CSNK2B* on CRC cell viability. Thus, inhibition of the *CSNK2B*-*mTOR* signaling may be effective strategy to suppress CRC progression. For example, 4,5,6,7-tetrabromobenzotriazole (TBB), a selective *CK2* inhibitor, has been reported to directly inhibit *CSNK2B* activity in vitro (Stengel et al. 2020), suggesting that it will be interesting to test whether TBB treatment can inhibit the biological functions of *CSNK2B* in CRC cells. Still, how *CSNK2B* activates the *mTOR* signaling pathway and its correlation with *CK2* protein, remain to be further addressed.

In summary, we identified *CSNK2B* as an oncogene in CRC, which promotes tumor cell proliferation in vitro and in vivo by activating the *mTOR* signaling pathway. Our findings indicated that *CSNK2B* might be a potential biomarker and therapeutic target for the diagnosis and treatment against CRC.

## Supplementary Information

Below is the link to the electronic supplementary material.

Supplementary file1 (TIF 18016 kb)

Supplementary file2 (TIF 14108 kb)

Supplementary file3 (TIF 4938 kb)

Supplementary file4 (TIF 3736 kb)

Supplementary file5 (TIF 3129 kb)
