# Supplementary figures and images for: CSNK2B contributes to colorectal cancer cell proliferation by activating the mTOR signaling
Source: J Cell Commun Signal. 2021 Apr 29;15(3):383–92. doi: 10.1007/s12079-021-00619-1 (PMC8222461; doi:10.1007/s12079-021-00619-1)

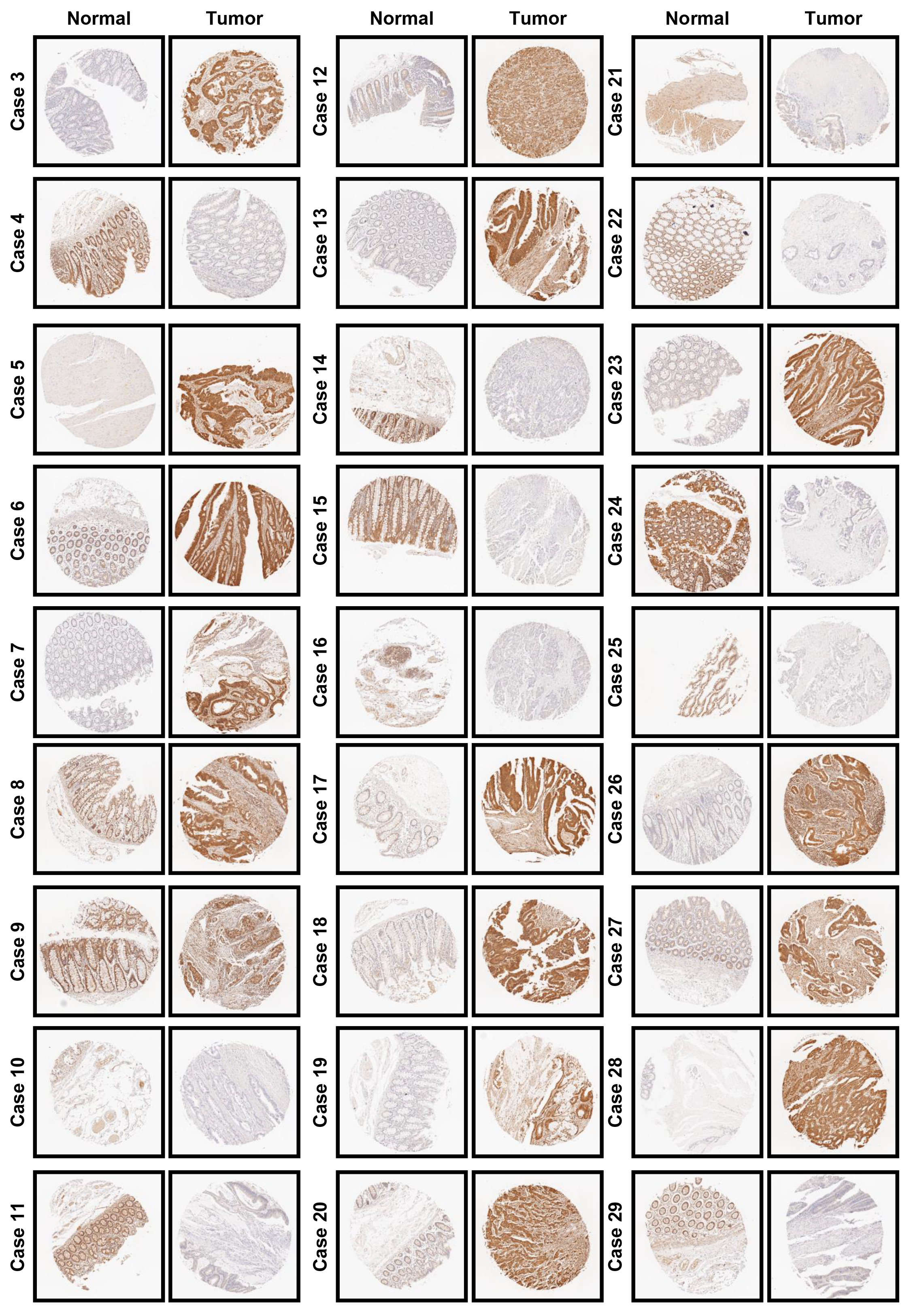

Supplement: Supplementary file 1 — Supplementary file1 (TIF 18016 kb) [file 12079_2021_619_MOESM1_ESM.tif]

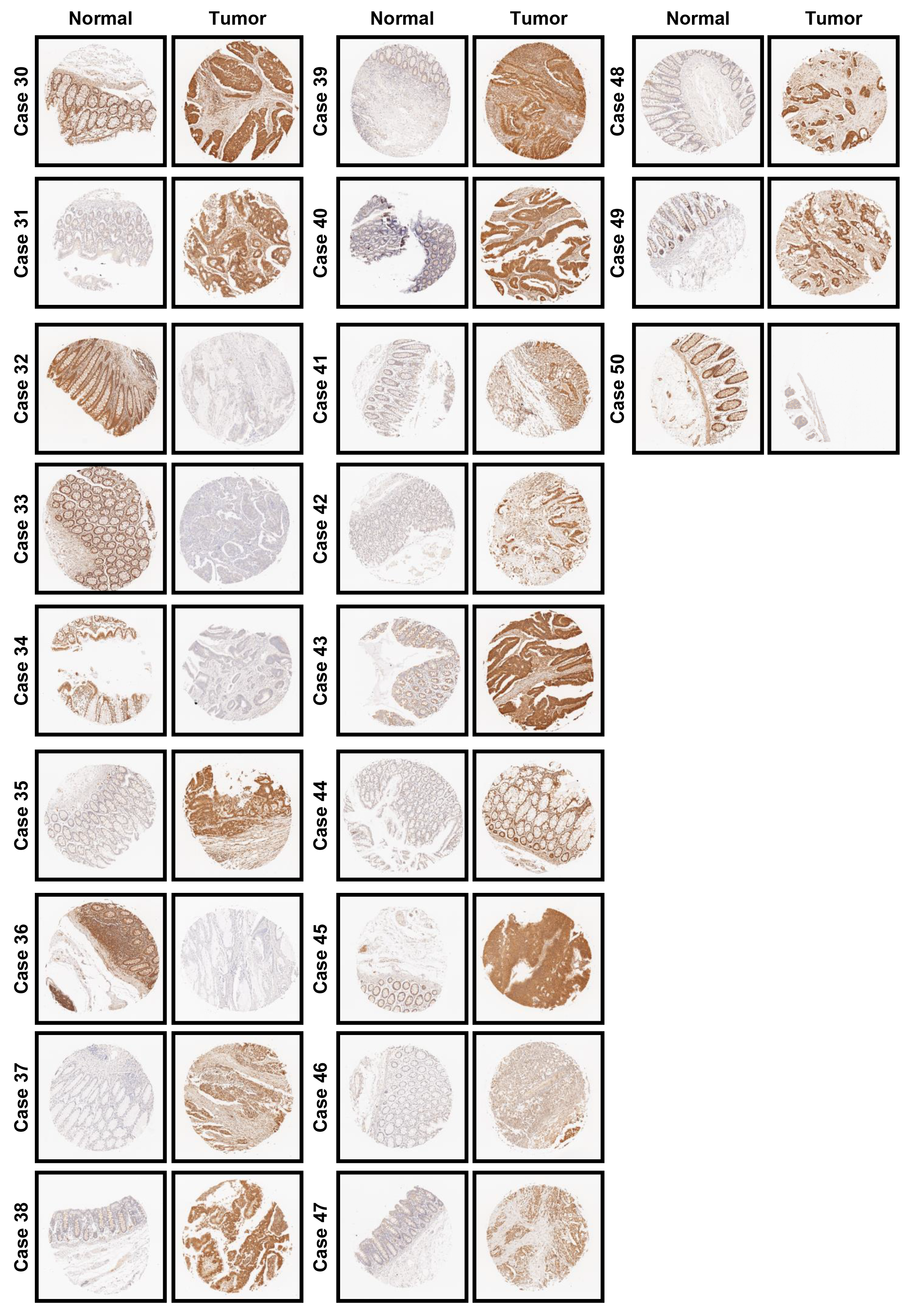

Supplement: Supplementary file 2 — Supplementary file2 (TIF 14108 kb) [file 12079_2021_619_MOESM2_ESM.tif]

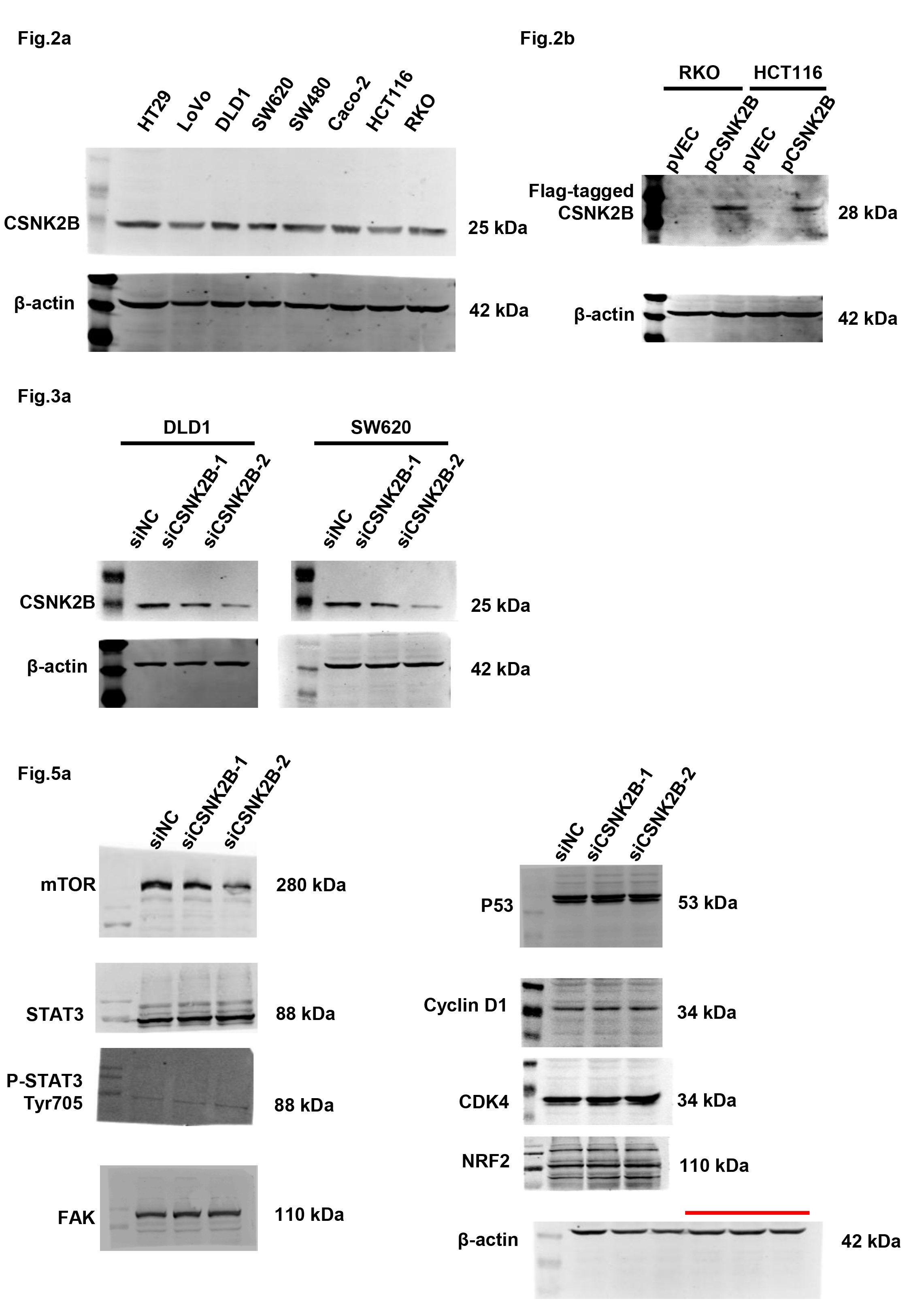

Supplement: Supplementary file 3 — Supplementary file3 (TIF 4938 kb) [file 12079_2021_619_MOESM3_ESM.tif]

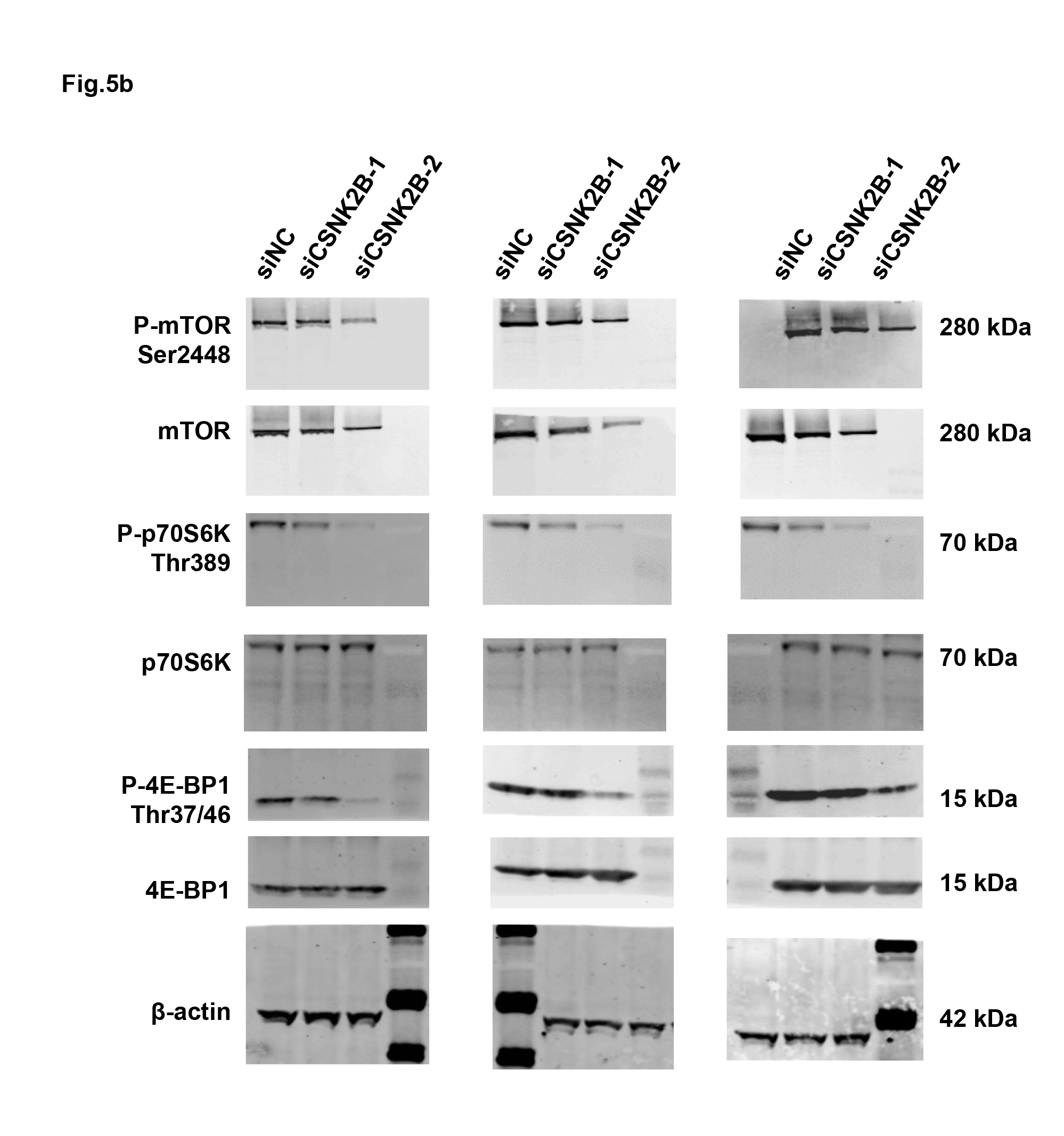

Supplement: Supplementary file 4 — Supplementary file4 (TIF 3736 kb) [file 12079_2021_619_MOESM4_ESM.tif]

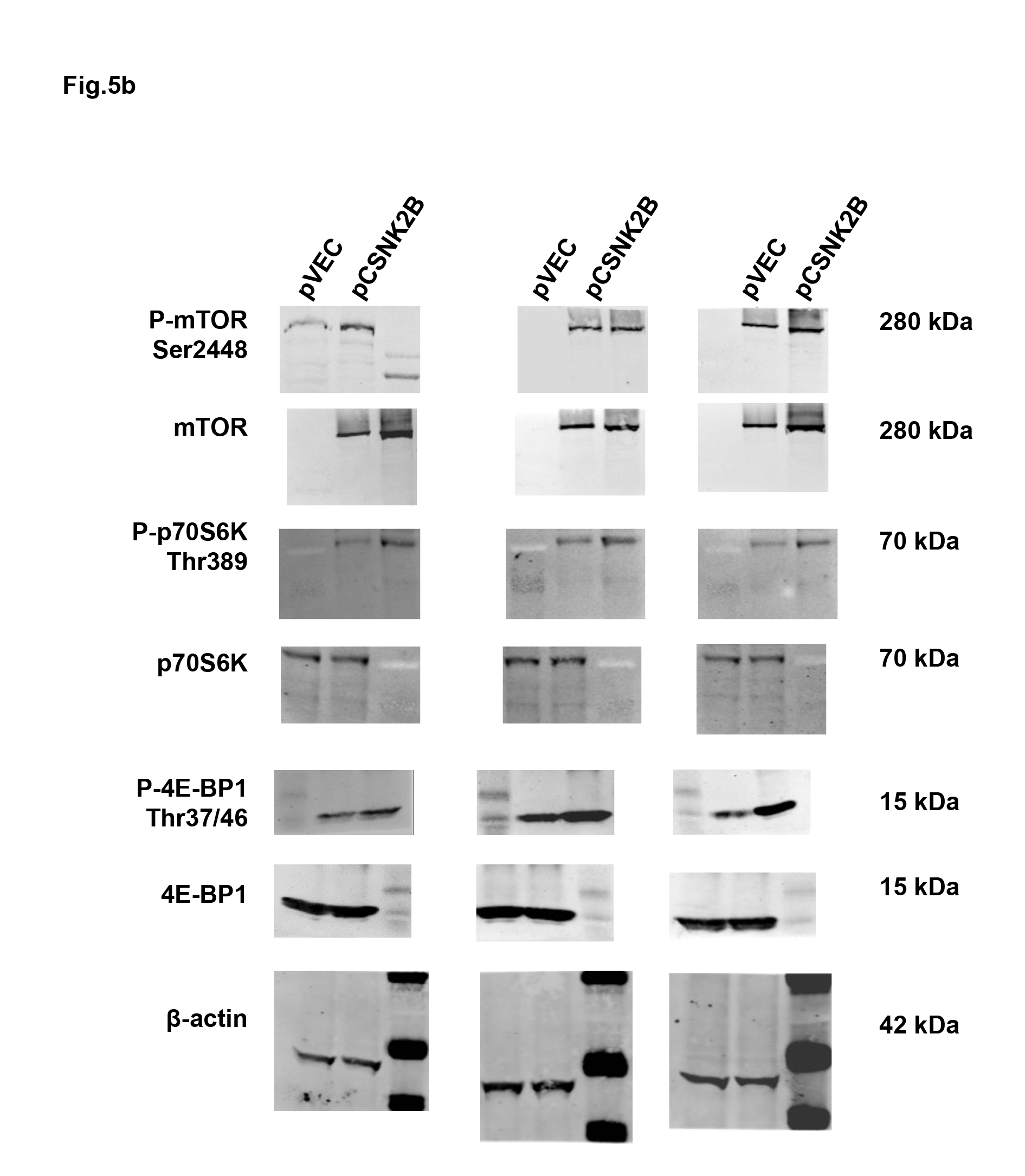

Supplement: Supplementary file 5 — Supplementary file5 (TIF 3129 kb) [file 12079_2021_619_MOESM5_ESM.tif]
